# Quality of care in patients with atrial fibrillation in primary care: a cross-sectional study comparing clinical and claims data

**DOI:** 10.3205/000240

**Published:** 2016-11-23

**Authors:** Rebekka Preuss, Jean-François Chenot, Aniela Angelow

**Affiliations:** 1 Department of Family Medicine, Institute for Community Medicine, University Medicine Greifswald, Germany

**Keywords:** oral anticoagulation, atrial fibrillation, primary health care, quality of health care, vitamin K antagonists & inhibitors

## Abstract

**Objectives: **Atrial fibrillation (AF) is a common cardiac arrhythmia with increased risk of thromboembolic stroke. Oral anticoagulation (OAC) reduces stroke risk by up to 68%. The aim of our study was to evaluate quality of care in patients with AF in a primary health care setting with a focus on physician guideline adherence for OAC prescription and heart rate- and rhythm management. In a second step we aimed to compare OAC rates based on primary care data with rates based on claims data.

**Methods:** We included all GP practices in the region Vorpommern-Greifswald, Germany, which were willing to participate (N=29/182, response rate 16%). Claims data was derived from the regional association of statutory health insurance physicians. Patients with a documented AF diagnosis (ICD-10-GM-Code ICD I48.-) from 07/2011–06/2012 were identified using electronic medical records (EMR) and claims data. Stroke and bleeding risk were calculated using the CHA_2_DS_2_-VASc and HAS-BLED scores. We calculated crude treatment rates for OAC, rate and rhythm control medications and adjusted OAC treatment rates based on practice and claims data. Adjusted rates were calculated including the CHA_2_DS_2_-VASc and HAS-BLED scores and individual factors affecting guideline based treatment.

**Results:** We identified 927 patients based on EMR and 1,247 patients based on claims data. The crude total OAC treatment rate was 69% based on EMR and 61% based on claims data. The adjusted OAC treatment rates were 90% for patients based on EMR and 63% based on claims data. 82% of the AF patients received a treatment for rate control and 12% a treatment for rhythm control. The most common reasons for non-prescription of OAC were an increased risk of falling, dementia and increased bleeding risk.

**Conclusion: **Our results suggest that a high rate of AF patients receive a drug therapy according to guidelines. There is a large difference between crude and adjusted OAC treatment rates. This is due to individual contraindications and comorbidities which cannot be documented using ICD coding. Therefore, quality indicators based on crude EMR data or claims data would lead to a systematic underestimation of the quality of care. A possible overtreatment of low-risk patients cannot be ruled out.

## Zusammenfassung

**Ziele:** Vorhofflimmern (VHF) ist eine häufige Herzrhythmusstörung, die mit einem erhöhten Risiko thromboembolischer Schlaganfälle einhergeht. Orale Antikoagulation (OAK) reduziert das Schlaganfallrisiko um bis zu 68%. Ziel unserer Studie war es, die Versorgungsqualität bei Patienten mit VHF unter dem Schwerpunkt leitliniengerechter OAK-Verordnung sowie Verordnung von Medikamenten zur Frequenz- und Rhythmuskontrolle zu untersuchen. Sekundäres Ziel war es, die direkt in der Praxis erhobenen Daten mit Abrechnungsdaten zu vergleichen.

**Methoden: **Alle hausärztlich tätigen Praxen aus der Region Vorpommern-Greifswald wurden um Studienteilnahme gebeten (N=29/182, Teilnahmerate 16%). Abrechnungsdaten wurden von der Kassenärztlichen Vereinigung Mecklenburg-Vorpommern zur Verfügung gestellt. Alle Patienten mit VHF (ICD-10-GM-Code ICD I48.-) von 07/2011–06/2012 wurden anhand der Praxissoftware und Abrechnungsdaten identifiziert. Schlaganfall- und Blutungsrisiko wurden anhand der CHA_2_DS_2_-VASc- und HAS-BLED-Scores berechnet. Es wurde eine rohe und eine adjustierte OAK-Verordnungsrate unter Berücksichtigung bestehender Kontraindikationen und individueller Faktoren leitliniengerechter Versorgung ermittelt.

**Ergebnisse: **Anhand der Praxisdaten wurden 927 Patienten und auf Basis der Abrechnungsdaten 1.247 Patienten mit VHF identifiziert. Die rohe OAK-Verordnungsrate betrug 69% basierend auf direkt in der Praxis erhobenen Daten und 61% basierend auf Abrechnungsdaten. Die adjustierte OAK-Verordnungsrate betrug 90% auf Basis der Praxisdaten und 63% auf Basis der Abrechnungsdaten. 82% der Patienten mit VHF erhalten eine medikamentöse Frequenz- und 12% eine Rhythmuskontrolle. Häufigste Gründe für die Nichtverordnung einer OAK waren Sturzgefährdung, Demenz und ein erhöhtes Blutungsrisiko.

**Schlussfolgerung:** Ein hoher Anteil der Patienten mit VHF wird leitliniengerecht antikoaguliert. Es liegt eine deutliche Diskrepanz zwischen roher und adjustierter OAK-Verordnungsrate vor. Diese ist auf eine ungenügende Abbildung individueller Kontraindikationen und Komorbiditäten in der ICD-Kodierung zurückzuführen. Aufgrund dieser Limitation führen Qualitätsindikatoren auf Basis von rohen Praxis- oder Routinedaten zu einer systematischen Unterschätzung der Leitlinienadhärenz. Eine mögliche Überversorgung im Bereich eines niedrigen Schlaganfallrisikos ist nicht auszuschließen.

## Introduction

Atrial fibrillation (AF) is the most common sustained cardiac arrhythmia [1], [2]. 5–10% of the over 60 years old and up to 15% of the 80 years old have AF. In about 30% of patients AF is asymptomatic [3]. AF is associated with a 5-fold increased risk of thromboembolic stroke and a 1.5-fold increased risk of fatal stroke [4], [5]. Oral anticoagulation (OAC) with vitamin K antagonists (VKA, e.g. phenprocoumon, warfarin) significantly reduces stroke risk by up to 68% [6]. Although OAC increases bleeding risk, it provides an overall benefit and was shown to be cost-effective [7], [8]. OAC is recommended by guidelines for patients with moderate to high risk of stroke and no contraindications. Additionally, the use of direct oral anticoagulants (DOACs) is increasing quickly, although there is still a lack of experience with long-term treatment [9], [10], [11], [12]. Antiplatelet agents are recommended for patients at low risk of stroke based on CHA_2_DS_2_-VASc score or with contraindications against OAC [11], [12]. 

An effective treatment with VKA requires INR (international normalised ratio) monitoring and an adjustment of the treatment regimen. Rate and rhythm management are further important aspects of AF treatment [4], [5].

International analyses and one German study using health claims data frequently show high rates of up to 50% of AF patients without OAC [13], [14], [15], [16], [17]. Physician related factors affecting OAC prescription have been investigated, but are difficult to assess using health claims data [18].

The aim of this study was to determine the quality of care in AF patients in primary care with focus on OAC treatment, reasons for non-prescription of OAC and rhythm-control treatment. In a second step we compared assessment of OAC rates based on data from practice records with health claims data.

## Materials and methods

### Study design and sample

This is a cross-sectional study based on clinical and claims-data from 29 general practitioners (GP). This sample was recruited by contacting all GPs (N=182) in the region Vorpommern-Greifswald. The mean age of the participating GPs was 51 years (SD 7), 62% were men and mean years practising was 15 years. Characteristics of participants corresponded to the average in Mecklenburg-Vorpommern (MV). The study was approved by the local Ethics Committee.

### Measurements

#### Clinical data

Study patients were identified using electronic medical records (EMR). All patients with at least one documented International Classification of Diseases (ICD-10) code I48.- between 07/2011–06/2012 were eligible for study inclusion. Patients seen as vacation coverage, with private health insurance and patients who died during the study period were excluded. Definite AF was defined if documented as a diagnosis in a hospital discharge letter or on ECG. Patients with documented successful cardioversion or catheter ablation were defined to have AF. Patients without hospital diagnosed AF and no AF on ECG were excluded from the study.

Pseudonymised data on age, sex, demographics, medication, INR-testing and co-morbidities for each patient were extracted by medical staff from the EMR and/or paper records using a standardized case report form. Anonymised copies of the most recent hospital discharge letters, ECG- and medication plan were obtained if available. Missing data was completed in an interview with the GP.

#### Claims data

Pseudonymised claims data used for billing was obtained from the regional association of statutory health insurance physicians (Kassenärztliche Vereinigung Mecklenburg-Vorpommern).

All patients with at least one ICD-code I48.- between 07/2011–06/2012 were eligible for the study. We excluded patients seen as vacation coverage and patients who died during the study period. For each patient, all diagnoses and procedures coded in ambulatory care were available.

A patient was defined to be on OAC, if one of the fee schedule items 32026 (prothrombin time), 32113 (plasma prothrombin time), 32114 (capillary blood prothrombin time), 32015 (oral anticoagulant therapy) or alternatively the ICD-10-GM-code Z92.1 (personal history of long-term (current) use of anticoagulants) was coded at least once in the study period. The schedule items were used as a proxy of an OAC treatment with a vitamin K antagonist.

### Data analysis

We calculated crude and adjusted OAC treatment rates based on clinical and claims data. Crude OAC rates were defined as:

***crude OAC rate_medical records_ = ***
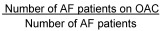


***crude OAC rate_claims data_ = ***
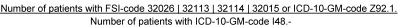


For claims data, only a calculation of treatment rates for VKA could be performed. Adjusted treatment rates were calculated based on clinical data according to guideline recommendations [4], [5]. OAC contraindications were defined based on bleeding risk (HAS-BLED score) and additional predefined criteria (Table 1 (Tab. 1)) [4], [5], [19], [20]. Intermittent contraindications for OAC (e.g. invasive procedures) were disregarded. We calculated adjusted OAC rates subtracting patients without OAC treatment but with a justified reason for non-prescription of OAC or patients with an alternative antithrombotic therapy according to the ESC guideline from the denominator [4], [5]: 

***adj OAC rate_medical records_ = ***
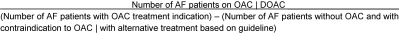


To calculate adjusted treatment rates for the claims data, we estimated individual stroke and bleeding risk using a modified CHA_2_DS_2_-VASc and HAS-BLED score adapted from Wilke et al. [13] (Table 2 (Tab. 2)). Patients with a CHA_2_DS_2_-VASc score ≤1 or HAS-BLED score ≥3 were subtracted from the denominator.

We calculated pooled and practice-specific crude and adjusted OAC rates and corresponding confidence intervals. Calculations were performed using SAS 9.3, SAS Institute Inc., Cary, NC, USA.

## Results

### Study population

We included 927 patients from 29 primary care practices (mean age 75 years, SD 10, 54% men) based on EMR and 1,247 patients (mean age 75 years, SD 10, 52% men) based on claims data in the study (Table 3 (Tab. 3)). The mean number of included patients per GP practice was 36 (SD 20, median 28) based on EMR and 48 (SD 33, median 43) based on claims data. 7% of the patients lived in a nursing home.

Main comorbidities included hypertension, vascular disease, diabetes mellitus and heart failure (Table 3 (Tab. 3)).

### Indication for OAC and risk of bleeding

The mean CHA_2_DS_2_-VASc score was 3.5 (SD 1.5) based on EMR and 3.9 (SD 1.7) based on claims data (Table 3 (Tab. 3)). 93% (860/927) of patients based on EMR and 91% (1130/1247) of patients based on claims data had an OAC indication (Table 3 (Tab. 3)).

The mean HAS-BLED score was 2.5 (SD 1.0) based on EMR and 1.0 (SD 0.6) based on claims data. An increased bleeding risk (HAS-BLED score ≥3) was found in 47% based on practice data and in 2% based on claims data.

**Figure 1:**
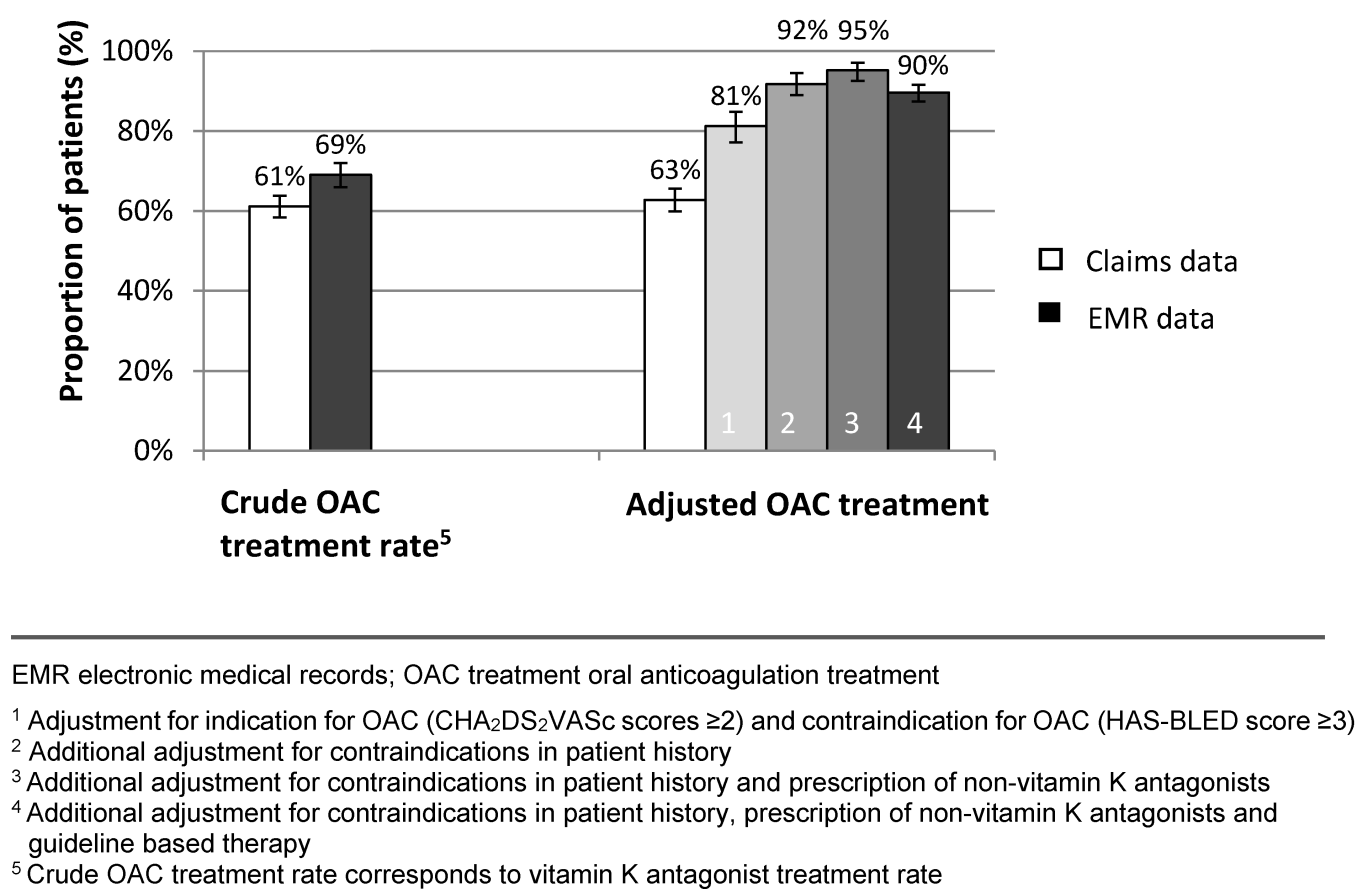
Mean crude and adjusted OAC treatment rates (pooled data)

**Figure 2:**
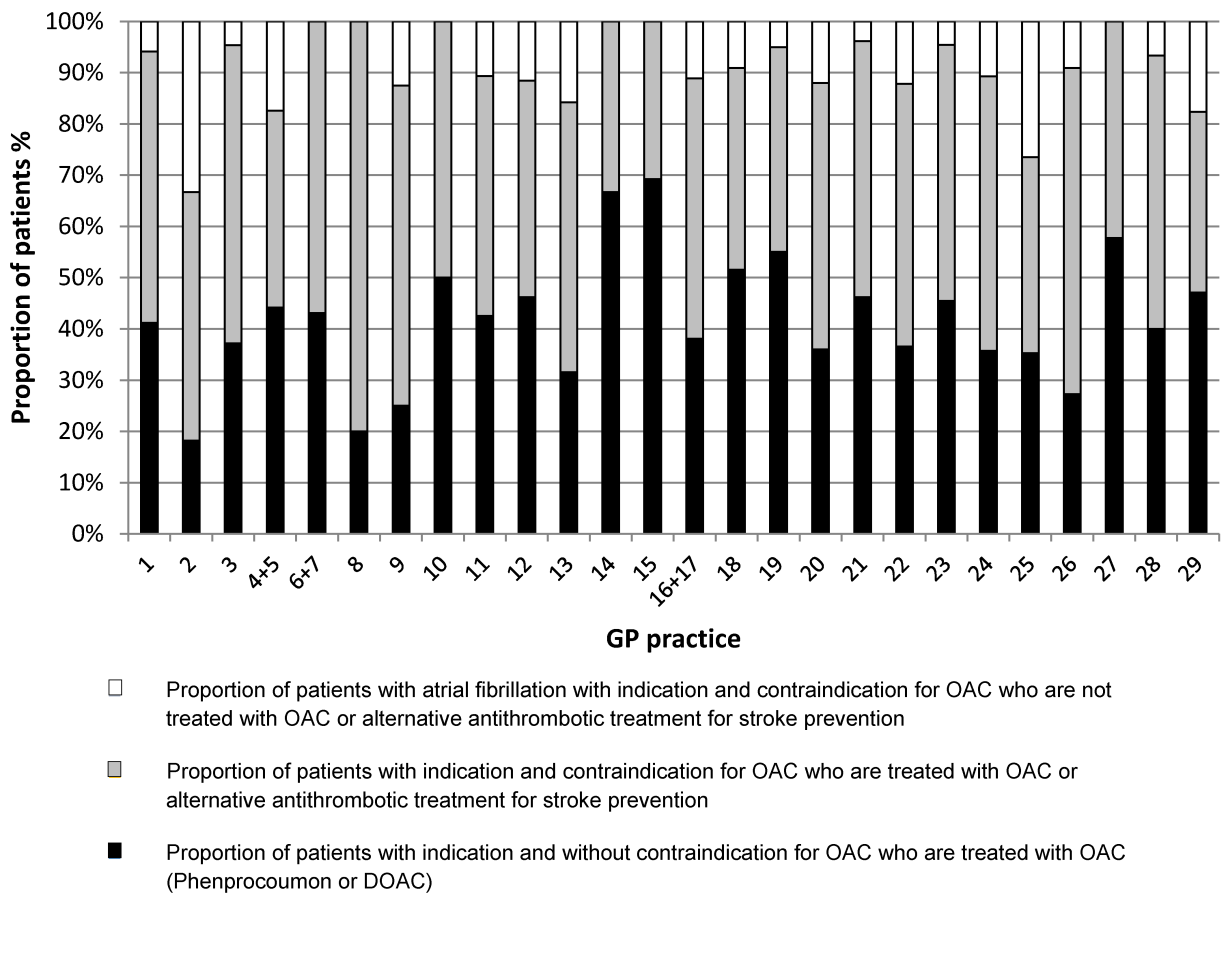
Proportion of study population with guideline based therapy (medical records)

Based on EMR, 69% (640/927) were treated with phenprocoumon, 5% (46/927) with a DOAC and 32% (294/927) with an antiplatelet agent or heparin. The crude OAC treatment rate was 69% (95% CI 65.0%–72.0%) (Figure 1 (Fig. 1)), corresponding to a crude OAC treatment rate of 71% (SD 17) per GP practice (Table 4, Figure 2 (Fig. 2)).

After correcting for diagnosis of a definite AF defined by the study criteria, guideline based OAC indication, bleeding risk, individual contraindications and alternative antithrombotic treatment, the adjusted mean OAC treatment rate was 90% (95% CI 87.4%–91.6%) (Figure 1 (Fig. 1)). The average adjusted OAC treatment rate was 91% (95% CI 87.4%–94.1%) per GP practice (Table 4, Figure 2 (Fig. 2)).

Based on claims data, the crude total OAC treatment rate was 61% (95% CI 58.3%–63.8%) (Figure 1 (Fig. 1)) and the average crude OAC treatment rate was 66% (SD 15) per GP practice. After taking into account a guideline based indication for OAC, individual contraindications and alternative antithrombotic treatment, 63% (95% CI 59.9%–65.6%) of patients received an OAC based on claims data (Figure 1 (Fig. 1)). This corresponds to an average OAC treatment rate of 67% (SD=15) per GP practice (Table 4 (Tab. 4), Figure 2 (Fig. 2)).

### Reasons for OAC underuse

The most common reasons for OAC underuse were increased risk of falling (23%, 67/287), dementia (19%, 54/287) and an increased risk of bleeding (15%, 43/287). Further reasons included tumors, social aspects, mental disorders, dialysis and history of cardioversion (2%, 6/287) or catheter ablation (2%, 7/287) as well as rare diseases affecting haemostasis.

### Rhythm and frequency control therapy

Based on EMR, 82% (758/927) of AF patients received at least one medication for rate control and 12% (110/927) at least one medication for rhythm control. Medications prescribed for rate control included betablockers (69%, 641/927), digitoxin (34%, 316/927), verapamil (3%, 25/927) and the I_f_-blocker ivabradine (0.1%, 1/927). Prescribed rhythm-control medications included amiodarone (5%, 43/927), dronedarone (3%, 23/927) or flecainide (4%, 40/927).

## Discussion

### Main findings

The present study assesses quality of care for AF patients in primary care. This is the first study in Germany to compare data from medical records with claims data in this patient group. Based on our analysis, a proportion of 90% of AF patients in primary care received a guideline concordant anticoagulation treatment after adjusting for reasons for non-prescription. Using claims data to calculate OAC rates underestimated the true proportion of correctly treated AF patients by 30%. 82% of AF patients received medication for rate control and 12% medication for rhythm control.

### Strengths and limitations

This is to our knowledge the first study in Germany comparing OAC based on clinical data with claims data. The response rate of 16% is comparable to other primary care studies [21], [22]. Characteristics of the participating practices corresponded well to the average in MV. It is probable, that GPs with interest in the topic were more likely to participate.

Patient characteristics corresponded well between practice and claims data. To minimize bias due to coding errors, AF diagnosis and comorbidities were evaluated using an interview with the GP and paper records. Data on comorbidities, obtained from the treating GP might be subject to observer bias.

Lower patient numbers and level of comorbidities identified in the claims data, suggest undercoding of AF in our study. To minimize bias due to undercoding, we applied a wider range of ICD codes to define comorbidities (Table 2 (Tab. 2)) [13]. Since we had no access to prescription data we could not adjust crude OAC rates based on claims data for alternative medications. Despite these limitations our results allow for the definition of a reference value regarding the proportion of OAC in AF patients based on clinical and claims data.

### OAC treatment

Three German studies using clinical data assessed OAC treatment rates. An OAC rate of 83% and a guideline concordant antithrombotic treatment rate of 93% were reported from the prospective German clinical registry ATRIUM [23]. An OAC treatment rate of 88% was found in a study comprising 361 AF patients from 45 primary care practices [24]. However, INR was estimated to be in the target range in only 56% of the treatment time. The study did not investigate alternative antithrombotic treatments. In contrast, a large prospective population based study reported a considerably lower OAC rate of 57% for antithrombotic therapy in 161 AF patients [25]. Only 5% (46/927) of AF patients in our study were treated with a DOAC. The expected rise in treatment rates poses a need to employ more detailed treatment data in future analyses. This data was not available in the claims data analysed for our study.

### OAC treatment indication and bleeding risk

In our study, more than 90% of AF patients were eligible for OAC based on a CHA_2_DS_2_-VASc score. The suitability of the CHA_2_DS_2_-VASc score for assessment of stroke risk and OAC indication has been controversially discussed and a recent Swedish study suggests overtreatment for low CHA_2_DS_2_-VASc score values [26], [27], [28], [29], [30].

47% (440/927) of AF patients in our study had an increased bleeding risk based on EMR, but only 15% based on the assessment of the treating physician. Several patient characteristics disfavouring OAC use (e.g. dementia) constitute an area of uncertainty with a need for individual shared decision making.

In our study, 2% (7/287) of patients did not receive an OAC after successful cardioversion or catheter ablation. Due to the high rate of recurrent AF and the lack of evidence of reduction in long term stroke risk, a continuation of OAC is now recommended [31], [32], [33], [34].

### Rate and rhythm control

The proportion of AF patients with rate control medication is a potential quality indicator. 18% (169/927) of AF patients in our study did not receive any medication for rate control, 69% (641/927) were treated with a beta blocker and 3% (25/927) with verapamil. However, beta blockers are recommended by guidelines as first line treatment, especially in patients with heart failure [5], [35]. Despite the narrow therapeutic range and lack of evidence for effects on relevant clinical outcomes, 34% (316/922) of AF patients were treated with digitalis glycosides. We hypothesise that patients received digitalis for a long period and treatment was not adapted to current recommendations [36]. One patient in our study was treated with ivabradine, although it is not licensed for AF treatment [37].

In our study, amiodarone (5%, 43/927), flecainide (4%, 40/927) or dronedarone (2/927 3%) were used for rhythm control. However, rhythm control has no clinical benefits compared to rate control in AF patients [34]. Amiodarone and dronedarone have relevant adverse effects, are not indicated in permanent AF and should be discontinued [4], [5]. A good effectiveness of flecainide was demonstrated for maintaining sinus rhythm after cardioversion especially in young patients without structural heart disease [38]. However, it is likely that in the majority of patients on long-term flecainide treatment (98%, 39/40) in our study, a re-evaluation is necessary because of known proarrhythmic effects.

OAC is currently recommended for AF patients with rate control medication and in patients under treatment with rhythm control medication and no stable sinus rhythm for at least 12 weeks [35], [37]. This was still controversially discussed at the time of the study.

### Comparison with claims data

A German study based on claims data of 183,448 AF patients, estimated that only 28% to 33% patient days were covered by OAC [13]. Prevalence rates for OAC or antithrombotic treatment were not reported. Our analysis shows that crude OAC rates based on claims data used for billing underestimate the true guideline adherence. This was mainly due to individual contraindications, which could not be documented using ICD codes. We estimate that an OAC rate of 70% based on EMR and 65% based on claims data corresponds to a very good adherence. An analysis strategy similar to the one employed in our study could be used for benchmarking purposes if applied to a representative sample (e.g. federal state level) and to develop a feedback instrument for individual practices. Full treatment information is currently only available in statutory health insurance data. Due to the high number of statutory health insurance funds (>50) and limited access to this data feedback at the individual practice level is therefore currently impossible.

## Conclusion

High rates of patients with AF receive a guideline concordant drug therapy. There is a considerable difference between crude and adjusted OAC treatment rates using EMR and claims data. This is mainly due to individual contraindications and uncoded comorbidities in EMR and claims data. Therefore, quality assessment based on crude claims data leads to an underestimation of the quality of care. According to our results, an OAC treatment rate of 60% to 70% based on claims data from the regional association of statutory health insurance physicians would be an appropriate target range for a quality indicator representing a satisfactory quality of care in a single practice. Our data indicates that drugs for rhythm control which so far have rarely been subject of quality measurements should be part of quality improvement activities. Readily available claims data could be used for feedback on practice level to improve quality of care. 

## Notes

### Competing interests

The authors declare that they have no competing interests.

### Financial support

The study was funded by the Central Research Institute of Ambulatory Health Care in Germany (Zentralinstitut für die kassenärztliche Versorgung in der Bundesrepublik Deutschland), Herbert-Lewin-Platz 3, 10623 Berlin, Germany.

### Acknowledgements

We are very grateful to Dr. med. Martin Sander, MD (Hausärztlicher Internist, Mercedes-Benz-Werk Kassel, Mercedesplatz 1, 34127 Kassel) for his valuable comments which helped us to develop the study design and for his contribution to the discussion of the results. 

We would like to thank all participating GP practices and the association of statutory health insurance physicians in Mecklenburg-Vorpommern for their support of the study.

## Figures and Tables

**Table 1:**
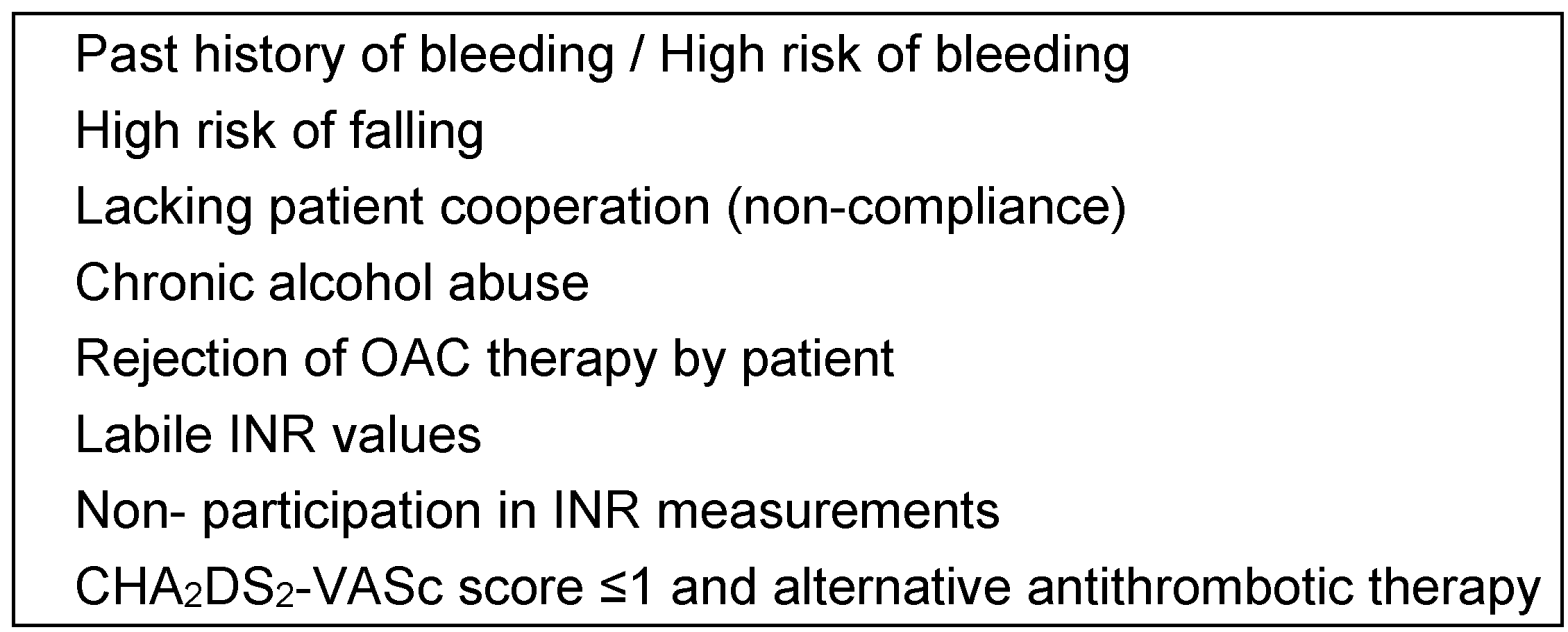
Absolute and relative contraindications against OAC treatment

**Table 2:**
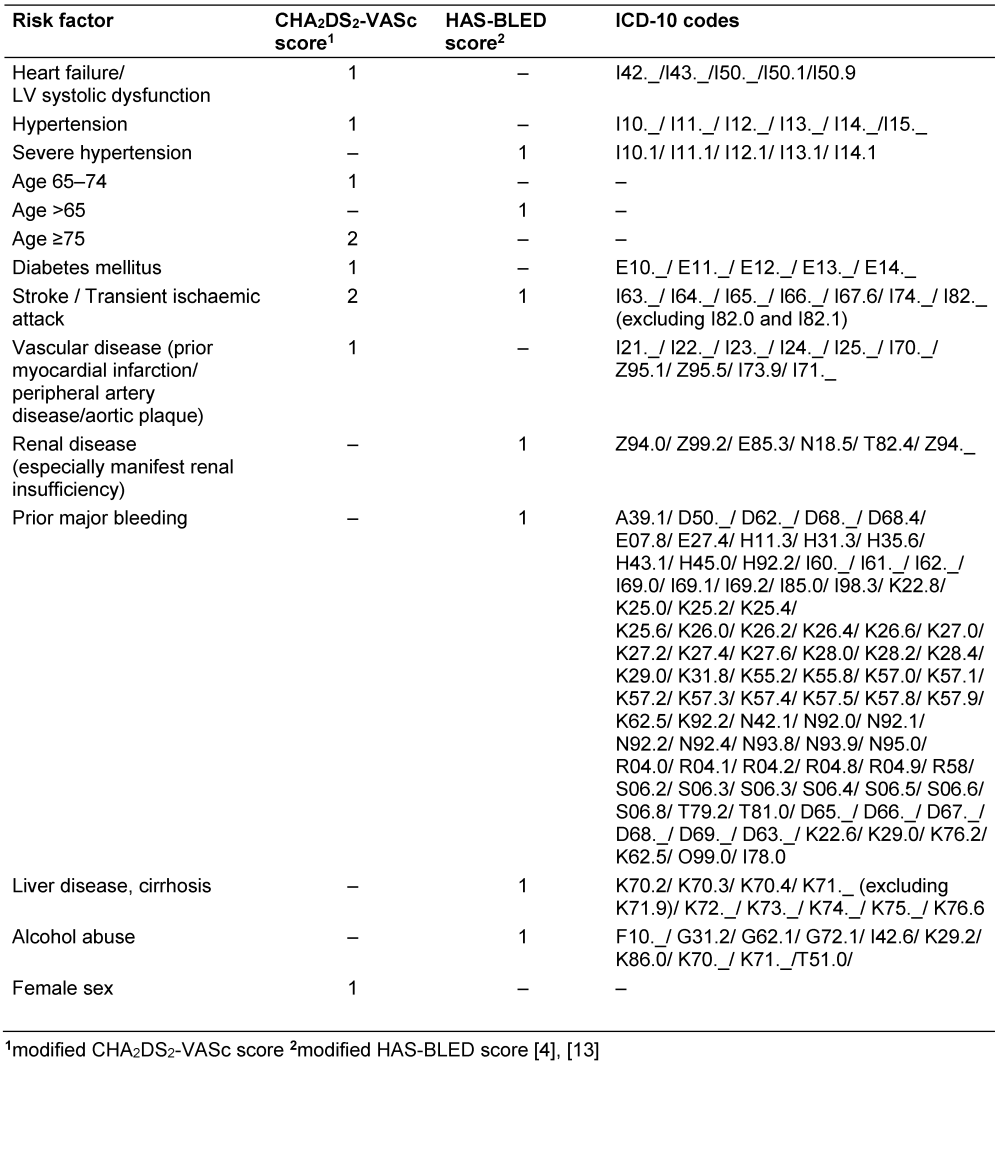
Criteria for the definition of stroke risk (modified CHA_2_DS_2_-Vasc score) and bleeding risk (modified HAS-BLED score)

**Table 3:**
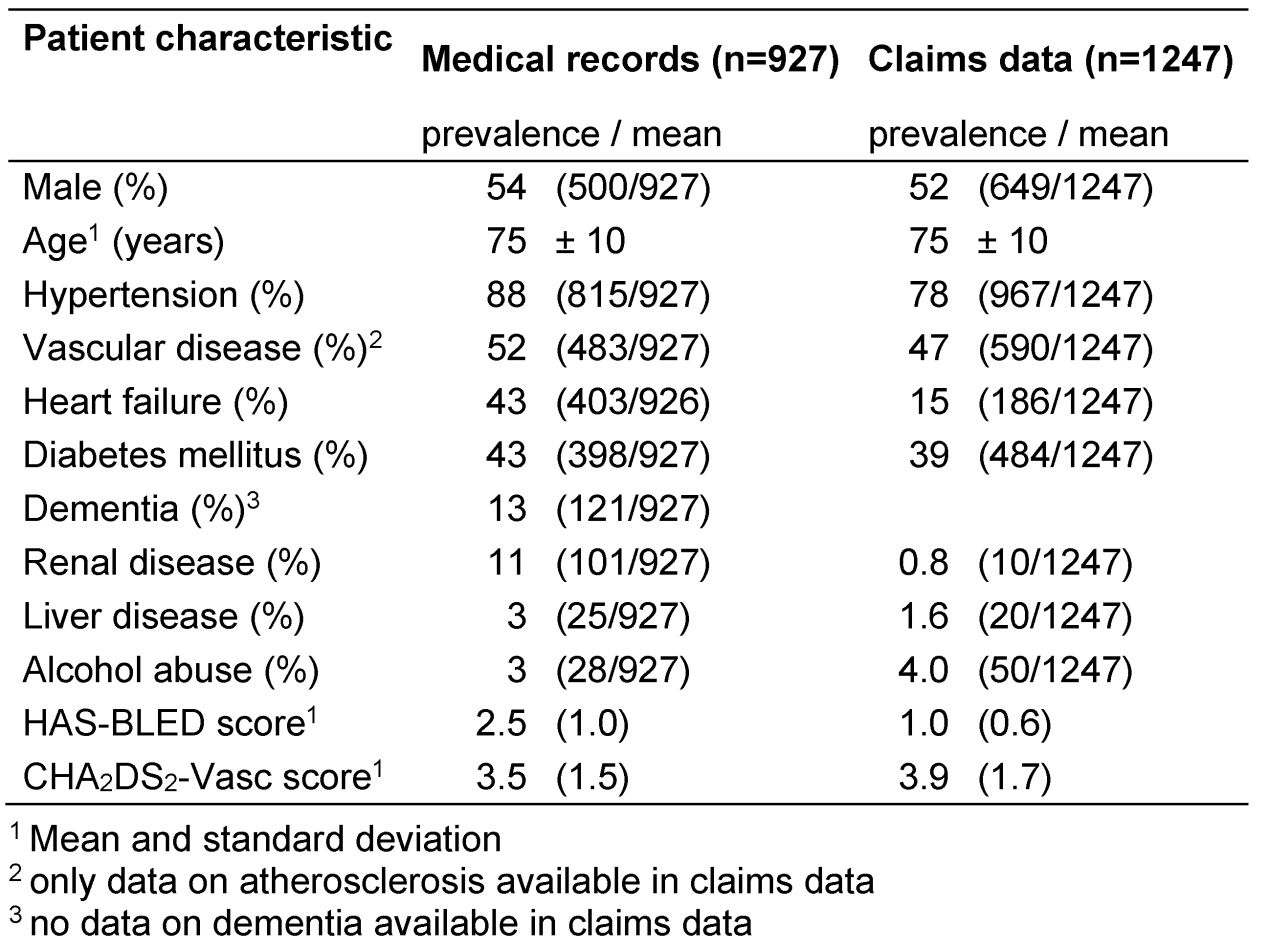
Characteristics of the study population

**Table 4:**
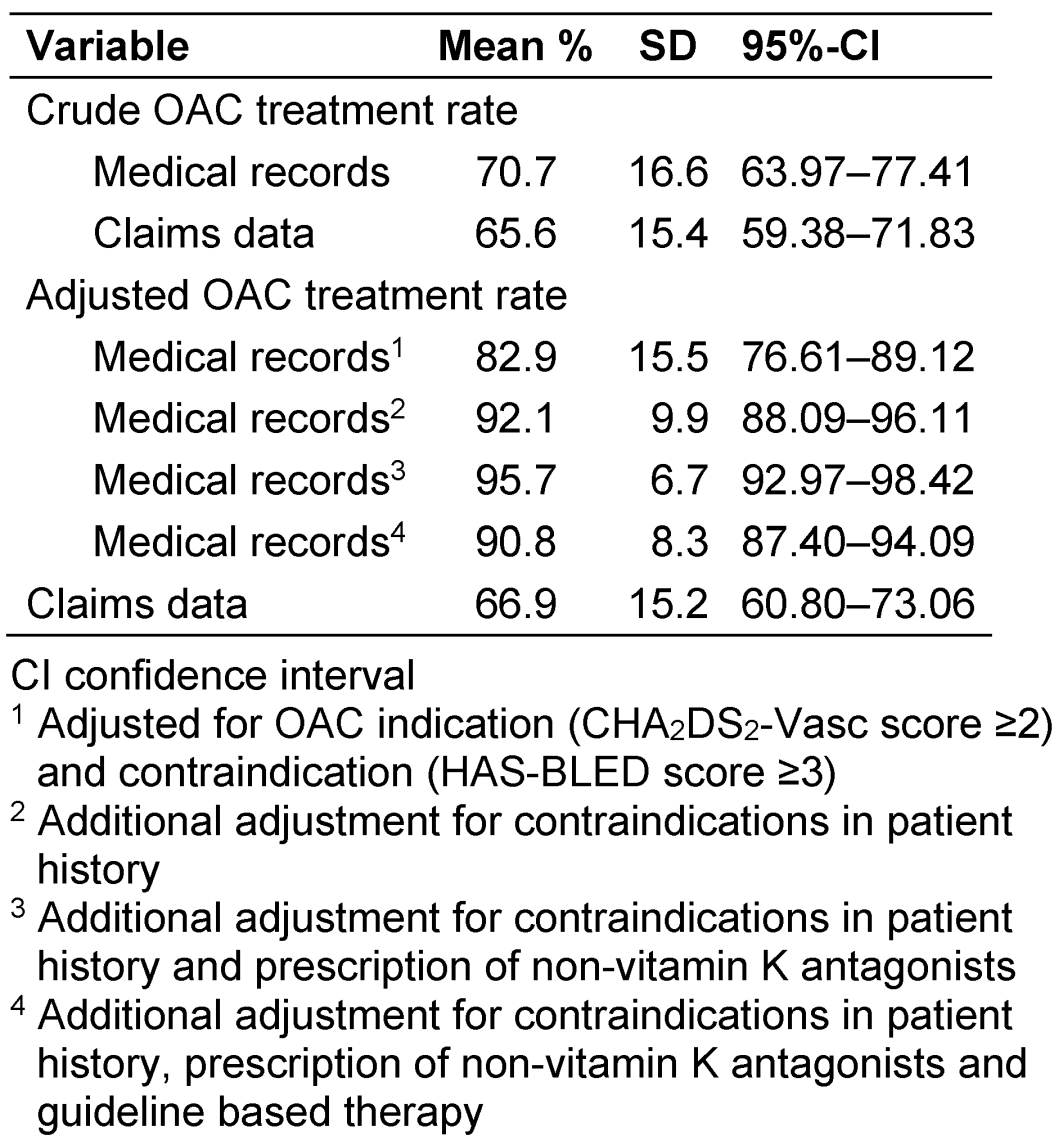
Mean crude and adjusted OAC treatment rates per GP practice

## References

[1] Heeringa J, van der Kuip DA, Hofman A, Kors JA, van Herpen G, Stricker BH, Stijnen T, Lip GY, Witteman JC (2006). Prevalence, incidence and lifetime risk of atrial fibrillation: the Rotterdam study. Eur Heart J.

[2] Naccarelli GV, Varker H, Lin J, Schulman KL (2009). Increasing prevalence of atrial fibrillation and flutter in the United States. Am J Cardiol.

[3] Page RL, Tilsch TW, Connolly SJ, Schnell DJ, Marcello SR, Wilkinson WE, Pritchett EL, Azimilide Supraventricular Arrhythmia Program (ASAP) Investigators (2003). Asymptomatic or "silent" atrial fibrillation: frequency in untreated patients and patients receiving azimilide. Circulation.

[4] Camm AJ, Kirchhof P, Lip GY, Schotten U, Savelieva I, Ernst S, Van Gelder IC, Al-Attar N, Hindricks G, Prendergast B, Heidbuchel H, Alfieri O, Angelini A, Atar D, Colonna P, De Caterina R, De Sutter J, Goette A, Gorenek B, Heldal M, Hohloser SH, Kolh P, Le Heuzey JY, Ponikowski P, Rutten FH (2010). Guidelines for the management of atrial fibrillation: the Task Force for the Management of Atrial Fibrillation of the European Society of Cardiology (ESC). Eur Heart J.

[5] Camm AJ, Lip GY, De Caterina R, Savelieva I, Atar D, Hohnloser SH, Hindricks G, Kirchhof P (2012). 2012 focused update of the ESC Guidelines for the management of atrial fibrillation: an update of the 2010 ESC Guidelines for the management of atrial fibrillation. Developed with the special contribution of the European Heart Rhythm Association. Eur Heart J.

[6] Hart RG, Pearce LA, Aguilar MI (2007). Meta-analysis: antithrombotic therapy to prevent stroke in patients who have nonvalvular atrial fibrillation. Ann Intern Med.

[7] Teng MP, Catherwood LE, Melby DP (2000). Cost effectiveness of therapies for atrial fibrillation. A review. Pharmacoeconomics.

[8] Bushnell CD, Matchar DB (2004). Pharmacoeconomics of atrial fibrillation and stroke prevention. Am J Manag Care.

[9] Arzneimittelkommission der deutschen Ärzteschaft (AkdÄ) Leitfaden der AkdÄ: Orale Antikoagulation bei nicht valvulärem Vorhofflimmern. Empfehlungen zum Einsatz der neuen Antikoagulantien Dabigatran (Pradaxa®) und Rivaroxaban (Xarelto®), Version 1.0, Sep 2012.

[10] De Caterina R, Husted S, Wallentin L, Andreotti F, Arnesen H, Bachmann F, Baigent C, Huber K, Jespersen J, Kristensen SD, Lip GY, Morais J, Rasmussen LH, Siegbahn A, Verheugt FW, Weitz JI, Coordinating Committee (2012). New oral anticoagulants in atrial fibrillation and acute coronary syndromes: ESC Working Group on Thrombosis-Task Force on Anticoagulants in Heart Disease position paper. J Am Coll Cardiol.

[11] Kirchhof P, Benussi S, Kotecha D, Ahlsson A, Atar D, Casadei B, Castella M, Diener HC, Heidbuchel H, Hendriks J, Hindricks G, Manolis AS, Oldgren J, Popescu BA, Schotten U, Van Putte B, Vardas P (2016). 2016 ESC Guidelines for the management of atrial fibrillation developed in collaboration with EACTS: The Task Force for the management of atrial fibrillation of the European Society of Cardiology (ESC)Developed with the special contribution of the European Heart Rhythm Association (EHRA) of the ESC Endorsed by the European Stroke Organisation (ESO). Eur J Cardiothorac Surg.

[12] Wann LS, Curtis AB, January CT, Ellenbogen KA, Lowe JE, Estes NA, Page RL, Ezekowitz MD, Slotwiner DJ, Jackman WM, Stevenson WG, Tracy CM (2011). 2011 ACCF/AHA/HRS focused update on the management of patients with atrial fibrillation (Updating the 2006 Guideline): a report of the American College of Cardiology Foundation/American Heart Association Task Force on Practice Guidelines. J Am Coll Cardiol.

[13] Wilke T, Groth A, Mueller S, Pfannkuche M, Verheyen F, Linder R, Maywald U, Kohlmann T, Feng YS, Breithardt G, Bauersachs R (2012). Oral anticoagulation use by patients with atrial fibrillation in Germany. Adherence to guidelines, causes of anticoagulation under-use and its clinical outcomes, based on claims-data of 183,448 patients. Thromb Haemost.

[14] Cohen N, Almoznino-Sarafian D, Alon I, Gorelik O, Koopfer M, Chachashvily S, Shteinshnaider M, Litvinjuk V, Modai D (2010). Warfarin for stroke prevention still underused in atrial fibrillation: Patterns of omission. Stroke.

[15] Baczek VL, Chen WT, Kluger J, Coleman CI (2012). Predictors of warfarin use in atrial fibrillation in the United States: a systematic review and meta-analysis. BMC Fam Pract.

[16] Portnoi VA (1999). The underuse of Warfarin treatment in the elderly. Arch Int Med.

[17] Weisbord SD, Whittle J, Brooks RC (2001). Is warfarin really underused in patients with atrial fibrillation?. J Gen Intern Med.

[18] Gross CP, Vogel EW, Dhond AJ, Marple CB, Edwards RA, Hauch O, Demers EA, Ezekowitz M (2003). Factors influencing physicians' reported use of anticoagulation therapy in nonvalvular atrial fibrillation: a cross-sectional survey. Clin Ther.

[19] Fumeaux T, Cornuz J, Polikar R, Blanc E, Junod A, Kappenberger L, Nicod P, Schläpfer J (2004). Guidelines for the clinical management of atrial fibrillation: a practical perspective. Swiss Med Wkly.

[20] Leitliniengruppe Hessen/PMV Forschungsgruppe (2009). Hausärztliche Leitlinie Antikoagulation mit Vitamin-K-Antagonisten Version 1.06.

[21] Bonevski B, Magin P, Horton G, Foster M, Girgis A (2011). Response rates in GP surveys - trialling two recruitment strategies. Aust Fam Physician.

[22] Hummers-Pradier E, Scheidt-Nave C, Martin H, Heinemann S, Kochen MM, Himmel W (2008). Simply no time? Barriers to GPs' participation in primary health care research. Fam Pract.

[23] Meinertz T, Kirch W, Rosin L, Pittrow D, Willich SN, Kirchhof P, ATRIUM investigators (2011). Management of atrial fibrillation by primary care physicians in Germany: baseline results of the ATRIUM registry. Clin Res Cardiol.

[24] McBride D, Brüggenjürgen B, Roll S, Willich SN (2007). Anticoagulation treatment for the reduction of stroke in atrial fibrillation: a cohort study to examine the gap between guidelines and routine medical practice. J Thromb Thrombolysis.

[25] Schnabel RB, Wilde S, Wild PS, Munzel T, Blankenberg S (2012). Atrial fibrillation: its prevalence and risk factor profile in the German general population. Dtsch Arztebl Int.

[26] Olesen JB, Lip GY, Hansen ML, Hansen PR, Tolstrup JS, Lindhardsen J, Selmer C, Ahlehoff O, Olsen AM, Gislason GH, Torp-Pedersen C (2011). Validation of risk stratification schemes for predicting stroke and thromboembolism in patients with atrial fibrillation: nationwide cohort study. BMJ.

[27] Olesen JB, Torp-Pedersen C, Hansen ML, Lip GY (2012). The value of the CHA2DS2-VASc score for refining stroke risk stratification in patients with atrial fibrillation with a CHADS2 score 0-1: a nationwide cohort study. Thromb Haemost.

[28] Coppens M, Eikelboom JW, Hart RG, Yusuf S, Lip GY, Dorian P, Shestakovska O, Connolly SJ (2013). The CHA2DS2-VASc score identifies those patients with atrial fibrillation and a CHADS2 score of 1 who are unlikely to benefit from oral anticoagulant therapy. Eur Heart J.

[29] Friberg L, Benson L, Rosenqvist M, Lip GY (2012). Assessment of female sex as a risk factor in atrial fibrillation in Sweden: nationwide retrospective cohort study. BMJ.

[30] Forslund T, Wettermark B, Wändell P, von Euler M, Hasselström J, Hjemdahl P (2014). Risks for stroke and bleeding with warfarin or aspirin treatment in patients with atrial fibrillation at different CHA(2)DS(2)VASc scores: experience from the Stockholm region. Eur J Clin Pharmacol.

[31] Chen HS, Wen JM, Wu SN, Liu JP (2012). Catheter ablation for paroxysmal and persistent atrial fibrillation. Cochrane Database Syst Rev.

[32] Van Gelder IC, Hagens VE, Bosker HA, Kingma JH, Kamp O, Kingma T, Said SA, Darmanata JI, Timmermans AJ, Tijssen JG, Crijns HJ, Rate Control versus Electrical Cardioversion for Persistent Atrial Fibrillation Study Group (2002). A comparison of rate control and rhythm control in patients with recurrent persistent atrial fibrillation. N Engl J Med.

[33] Verma A, Champagne J, Sapp J, Essebag V, Novak P, Skanes A, Morillo CA, Khaykin Y, Birnie D (2013). Discerning the incidence of symptomatic and asymptomatic episodes of atrial fibrillation before and after catheter ablation (DISCERN AF): a prospective, multicenter study. JAMA Intern Med.

[34] Wyse DG, Waldo AL, DiMarco JP, Domanski MJ, Rosenberg Y, Schron EB, Kellen JC, Greene HL, Mickel MC, Dalquist JE, Corley SD, Atrial Fibrillation Follow-up Investigation of Rhythm Management (AFFIRM) Investigators (2002). A comparison of rate control and rhythm control in patients with atrial fibrillation. N Engl J Med.

[35] Chatterjee S, Biondi-Zoccai G, Abbate A, D'Ascenzo F, Castagno D, Van Tassell B, Mukherjee D, Lichstein E (2013). Benefits of β blockers in patients with heart failure and reduced ejection fraction: network meta-analysis. BMJ.

[36] Gheorghiade M, Fonarow GC, van Veldhuisen DJ, Cleland JG, Butler J, Epstein AE, Patel K, Aban IB, Aronow WS, Anker SD, Ahmed A (2013). Lack of evidence of increased mortality among patients with atrial fibrillation taking digoxin: findings from post hoc propensity-matched analysis of the AFFIRM trial. Eur Heart J.

[37] Martin RI, Pogoryelova O, Koref MS, Bourke JP, Teare MD, Keavney BD (2014). Atrial fibrillation associated with ivabradine treatment: meta-analysis of randomised controlled trials. Heart.

[38] Aliot E, Capucci A, Crijns HJ, Goette A, Tamargo J (2011). Twenty-five years in the making: flecainide is safe and effective for the management of atrial fibrillation. Europace.

